# Low-level cadmium exposure induced hormesis in peppermint young plant by constantly activating antioxidant activity based on physiological and transcriptomic analyses

**DOI:** 10.3389/fpls.2023.1088285

**Published:** 2023-01-23

**Authors:** Bin Wang, lvna Lin, Xiao Yuan, Yunna Zhu, Yukun Wang, Donglin Li, Jinming He, Yanhui Xiao

**Affiliations:** ^1^ Guangdong Provincial Key Laboratory of Utilization and Conservation of Food and Medicinal Resources in Northern Region, Shaoguan University, Shaoguan, China; ^2^ Henry Fok College of Biology and Agriculture, Shaoguan University, Shaoguan, China; ^3^ Shaoguan Aromatic Plant Engineering Research Center, Shaoguan University, Shaoguan, China; ^4^ College of Horticulture, South China Agricultural University, Guangzhou, China

**Keywords:** cadmium stress, hormesis effect, transcriptomic analysis, antioxidant system, peppermint plant

## Abstract

As one of the most toxic environmental pollutants, cadmium (Cd) has lastingly been considered to have negative influences on plant growth and productivity. Recently, increasing studies have shown that low level of Cd exposure could induce hormetic effect which benefits to plants. However, the underlying mechanisms of Cd-triggered hormesis are poorly understood. In this study, we found that Cd stress treatment showed a hormetic effect on peppermint and Cd treatment with 1.6 mg L^-1^ concertation manifested best stimulative effects. To explore the hormesis mechanisms of Cd treatment, comparative transcriptome analysis of peppermint young plants under low (1.6 mg L^-1^) and high (6.5 mg L^-1^) level of Cd exposure at 0 h, 24 h and 72 h were conducted. Twelve of differentially expressed genes (DEGs) were selected for qRT-PCR validation, and the expression results confirmed the credibility of transcriptome data. KEGG analysis of DEGs showed that the phenylpropanoid biosynthesis and photosynthesis were important under both low and high level of Cd treatments. Interestingly, GO and KEGG analysis of 99 DEGs specifically induced by low level of Cd treatment at 72 h indicated that these DEGs were mainly involved in the pathway of phenylpropanoid biosynthesis and their functions were associated with antioxidant activity. The expression pattern of those genes in the phenylpropanoid biosynthesis pathway and encoding antioxidant enzymes during 72 h of Cd exposure showed that low level of Cd treatment induced a continuation in the upward trend but high level of Cd treatment caused an inverted V-shape. The changes of physiological parameters during Cd exposure were highly consistent with gene expression pattern. These results strongly demonstrate that low level of Cd exposure constantly enhanced antioxidant activity of peppermint to avoid oxidative damages caused by Cd ion, while high level of Cd stress just induced a temporary increase in antioxidant activity which was insufficient to cope with lasting Cd toxicity. Overall, the results presented in this study shed a light on the underlying mechanisms of the Cd-mediated hormesis in plant. Moreover, our study provided a safe method for the efficient utilization of mild Cd-contaminated soil as peppermint is an important cash plant.

## Introduction

Farmland cadmium (Cd) pollution is a universal issue in many countries especially developing countries (Yuan et al., 2021). For example, it has been reported that Cd-contaminated soil covered an area of more than 20% of the total farmland area in China (Tang et al., 2016; Xie et al., 2021). Cd is one of the most toxic environmental pollutants with a long biological half-life (Ismael et al., 2019; Yu et al., 2022), which is concurred to give rise to continuously harmful effects for farmland soil (Zhang et al., 2021). As a result, Cd ion can finally enter into the human body through a contaminated food chain, due to its high mobility from soil to plant and thus causing a Cd accumulation in plant tissues (Clabeaux et al., 2011; Zhu et al., 2018). The dietary ingestion of Cd-polluted foods causes severe Cd toxicity for the human body, including growth inhibition and some serious diseases (e.g. cardiovascular diseases, renal and gut and liver damages, and even some cancers) (Jomova and Valko, 2011; Satarug et al., 2011; Zhang and Reynolds, 2019; Lemaire et al., 2020). Therefore, the remediation and utilization of Cd-contaminated farmland soil *via* safe methods is an urgent and hard task, which is distinguished from the remediation of other Cd-polluted soils such as mine and urban soils, because it directly concerns the security and quality of agricultural products.

Peppermint (*Mentha piperita*) is an oil producing plant from the *Lamiaceae* family (Mahendran and Rahman, 2020). Previous studies have been reported that peppermint plant has the ability to adapt and/or resist Cd stress (Zheljazkov et al., 2006; Demrezen and Aksoy, 2010). Essential oil is the major products of peppermint, which has wide applications in the food and pharmaceutical and chemical industries (Tholl, 2015). The extraction of essential oil without metals including Cd from aromatic plant can be readily achieved by using some special techniques such as steam distillation (Zheljazkov et al., 2006; Gautam and Agrawal, 2017). Such reports indicate that planting aromatic plants including peppermint may be a safe method for the utilization of Cd-contaminated soils (Gautam and Agrawal, 2017). However, numerous studies reported that Cd exposure had inhibitory effects on the growth of most plants even under very low concentration, and thus resulting in a reduced biomass (Liu et al., 2018; Michelle et al., 2019; Halim et al., 2020; Zhu et al., 2021), which seriously restricts the yield of essential oil. Therefore, it is essential to know whether and how different Cd exposure levels influence the growth of peppermint plant.

The hormesis, a phenomenon improving plant life performance under low Cd dosage, has been found in an increasing number of plant species including some aromatic/medicinal plants (Carvalho et al., 2020). For example, a low level (25 mg kg^-1^) of Cd exposure promoted the growth and essential oil yield of sweet basil, but Cd treatments with high dosage resulted in adverse effects (Prasad et al., 2011). In addition, the yield and quality of essential oil in lemongrass were improved when the seedlings were grown on a heavy-metal-contaminated soil in which Cd concentration was close to 50 mg kg^-1^, but that was reduced when Cd concentration was exceeded 50 mg kg^-1^ (Gautam and Agrawal, 2017). Moreover, Cd exposure treatments at Cd concertation varying from 0.37 mg kg^-1^ to 7.39 mg kg^-1^ promoted the growth performance of *Polygonatum sibiricum*, a traditional Chinese medicinal herb, with elevations in tuber biomass and plant height, and Cd stress treatment under 0.37 mg kg^-1^ concertation had the best stimulative effects on plant growth (Xie et al., 2021). And therefore, the authors proposed that planting medicinal plants might be a safe way for efficient utilization of low Cd-contaminated farmland (Xie et al., 2021). Although Cd-induced hormesis is observed in many plant species, the mechanisms mediated are poorly understood.

Cd stress with high degree usually cause oxidative stress for plant cells, because Cd ion can easily trigger excessive synthesis of reactive oxygen species (ROS) or result in failure in scavenging ROS, which will inevitably cause oxidative damages for the intracellular biomacromolecules such as protein, lipid as well as nucleotide (Yuan et al., 2013). Currently, it is suggested that Cd-induced hormesis is closely related to the activation of antioxidant defense system in plant (Jia et al., 2015; Muszyńska et al., 2018; Carvalho et al., 2020; Makowski et al., 2020). For instance, the exposure of *Arabidopsis thaliana* seedlings to a short-term Cd stress significantly induced the activities of ascorbate peroxidase (APX), catalase (CAT) and glutathione reductase (GR) (Carneiro et al., 2017). The low level of Cd stress treatment induces the accumulation of responded substances, such as anthocyanins, flavonoids, phenolics, ascorbic acid and other free organic acids (Vinogradova and Glukhov, 2021), they are known non-enzymatic antioxidants in plants. These reports suggest that improving antioxidant activity to scavenge ROS is highly associated with hormetic effects in plant, and its mechanism investigation is of great significance. However, the activation mode of antioxidant system induced by Cd stress remains unclear. Firstly, which concentrations of Cd treatment are antioxidants induced: all Cd treatments? Secondly, are they activated in a transient manner or in a sustained manner? Thirdly, at what levels are different antioxidants induced: protein or gene level, or both? Fourthly, how are different antioxidants induced: all at the same time or in a specific pattern?

The objective of this study was to explore Cd-induced hormesis effects and to reveal the possible mechanisms involved in that through analyzing physiological and transcriptomic responses of peppermint young plants in response to different degrees of Cd stress. The results presented in this work would provide helpful information for understanding the mechanisms underlying Cd-induced hormesis effects. Moreover, this study put forward a safe and efficient way for the utilization of Cd-contaminated farmland soil.

## Materials and methods

### Plant materials and experimental design

Peppermint (*Mentha piperita* cv hengjing gaoyou) from the variety of “Shanghai 9” is an excellent clone with strong lodging resistance and high essential oil yield, which has a huge advantage for the industrial application.

Rooted cuttings of peppermint were employed to investigate the hormesis effects induced by Cd treatment in this study. Peppermint young plants were cultured by using the method of cuttage at Shaoguan university in March 2019 in Shaoguan, China. 1-week-old rooted cuttings were moved to a vessel and incubated within 1/2 Hoagland solution for one week for acclimatization. Young plants were grown in hydroponics containing Hoagland solution supplemented with different concentrations of Cd ion (CdCl_2_, Aladdin, China). The final concentrations of Cd ion in each treatment are as follows: 0 mg L^-1^ (control), 0.8 mg L^-1^, 1.6 mg L^-1^, 3 mg L^-1^ or 6.5 mg L^-1^, respectively. During different concentrations of Cd stress treatment, peppermint leaves were collected at 0 h, 24 h and 72 h for further investigations. Each treatment was performed three times, and contained a total of 54 clones (18 clones for each biological replicate), and 6 clones from three replicates were collected at each sampling point. Harvested leaf samples were quickly frozen with liquid nitrogen and stored at -80 °C for subsequent experiments.

### Determination of plant growth performance

The growth performance of peppermint young plants was assessed when the rooted cuttings were exposed in different concentrations of Cd solutions for 7 days. Before assessment, root system of fresh young plants was washed with tap water and soaked into a CaCl_2_ solution (10 mmol L^-1^) to remove and dissolve the residual Cd. The roots were washed for three times. Residual moisture on the surface of young plants was removed using clean tissues. The plant height was determined using a dividing rule and the number of lateral branches was recorded. The root system of peppermint young plants was scanned using a MICROTEK scanner (MRS-9600 TFU2L, China) following a standard flow. The fresh and dry weight of leaves, shoots and roots were respectively evaluated using an electronic balance. Before the measurement of dry weight, peppermint organs were dried to a constant weight in an oven (at 70 °C). Each measurement was carried out three times using different samples.

### Hormetic analysis of Cd treatments

Hormetic analysis for fresh weight response variable of the whole plant of peppermint young plants was performed with the model developed by Brain and Cousens (1989).

### RNA extraction, cDNA library construction and Illumina sequencing

Total RNA of peppermint samples was extracted using a RNAprep Pure Plant Plus Kit (Tiangen, China) basing on the manufactory’s instructions. The concentrations of total RNA were measured with a NanoDrop 2000 spectrophotometer (Thermo, USA). The purity and integrity of RNA were checked by an Agilent Bioanalyzer 2100 system (Agilent Technologies, USA) and 1.0% denaturing agarose gels, respectively. The cDNA library construction and RNA sequencing were performed in the Biomarker Biotechnology Corporation (Beijing, China). A total of 21 cDNA libraries were constructed using an Illumina TruSeqTM RNA Sample Preparation Kit (Illumina, USA) following the manufacturer’s recommendations. All libraries were sequenced using the Illumina system HiSeq2500 following the standard procedure. The detailed processes of cDNA library construction and RNA sequencing were described in our former study (Wang et al., 2021).

### Transcriptome assembly and gene functional annotation

Clean reads were generated after the removal of adapter contaminations and trimming nucleotides with low quality-score. Then the clean reads were processed for assembly by Trinity software (Grabherr et al., 2011). The clean reads of each sample with high quality were mapped to unigenes using HISAT (Hierarchical indexing for spliced alignment of transcripts) method (Kim et al., 2015). The mapped reads were used in the following analysis.

Finally, all unigenes were aligned against various public databases, including NR (non-redundant protein database of the National Center for Biotechnology Information), Swiss-Prot protein (http://www.uniprot.org/), COG (Clusters of Orthologous Groups) (http://www.ncbi.nlm.nih.gov/COG/), KOG (eu-Karyotic Orthologous Groups), GO (Gene Ontology) (http://www.geneontology.org/) and KEGG (Kyoto Encyclopedia of Genes and Genomes) (http://www.genome.jp/kegg) databases, to annotate the specific gene function using BLAST program (E value < 10^-5^) (Wang et al., 2021). The GOseq R packages were employed to perform GO enrichment analysis (Ashburner et al., 2000; Zhu et al., 2018). KEGG pathway enrichment analysis was performed using KOBAS software (Kanehisa et al., 2019). GO and KEGG analysis were performed using BMKCloud (www.biocloud.net).

### Identification of differentially expressed genes (DEGs)

Gene expression values were presented with Fragments Per kilobase per Million reads (FPKM) by the RSEM software (Li and Dewey, 2011). The differences of gene expression values were compared between treatments and the control by calculating FPKM (Zhang et al., 2020). Genes with fold change (FC) of expression levels ≥1.5 and q-value (adjusted P-value) ≤0.01 were considered as significant differentially expressed genes (DEGs) (Wang et al., 2021).

### Validation of transcriptomic data by qRT-PCR

Twelve DEGs involved in Cd stress response were randomly selected for qRT-PCR. Total RNA of peppermint leaves was extracted using a RNAprep Pure Plant Plus Kit (Tiangen, China). Subsequently, total RNA was treated with DNaseI to remove residual DNA, and then the cDNA synthesis was performed using a 1st Strand cDNA Synthesis Kit (Yeasen, China). qRT-PCR analysis was carried out on a CFX96 Real-Time PCR Detection System (Bio-Rad, USA) with the following program: 95°C for 1 min, followed by 40 cycles at 95°C for 15 s, 58°C for 15 s, and 72°C for 30 s.

The relative expression levels of the twelve genes were calculated with the 2^-ΔΔCT^ method (Livak and Schmittgen, 2001; Schmittgen and Livak, 2008). The *actin* gene (GenBank: KR082011.1) was used as an internal control gene to perform normalization. The expression patterns of *actin* in different samples were shown in **Supplementary Figure 1**. The results indicated that the expression of this reference gene was not significantly changed among different Cd-treated samples and the control, demonstrating that the expression of this gene is stable before and after Cd treatment. The specific primers of genes for qRT-PCR were designed using the Primer-BLAST of NCBI and were listed in **Supplementary Table 1**.

### Determination of total phenol and total flavonoid contents in peppermint

Total phenolic content (TPC) in peppermint leaves was determined using the method of Folin-Ciocalteu (Ainsworth and Gillespie, 2007). 1.0 g of fresh leaf samples and 5 mL of 1% (v/v) HCl-methanol reagent were well homogenized with a 15 mL of centrifuge tube using a handheld homogenizer. The homogenates were held at ambient temperature (25 °C) for 3 hours and then were centrifuged at 4 °C (12,000 × g) for 15 min. For the measurement of TPC, 0.5 mL of supernatants, 1.5 mL of sodium carbonate (100 mM), and 1.0 mL of Folin-Ciocalteu reagent were well mixed in a new tube and reacted at 25°C for 30 min in the dark. After 30 min of reaction, the absorbance was determined at 765 nm using an UV-visible spectrophotometer (MAPADA UV-1800, China). The TPC was calculated based on a standard curve of gallic acid (GA) and the results were expressed as milligram GA equation per kilogram of fresh weight (mg kg^-1^ FW).

For the measurement of total flavonoid content (TFC), total flavonoid in peppermint leaves was first extracted with 10 mL of acetic acid and acetone mixing solution (0.5:70, v/v) at 4 °C for 2 hours with minor revisions (Valcarcel et al., 2015). Then, 1.5 mL of supernatants, 1.0 mL of distilled water, 0.1 mL of 10% (w/v) AlCl_3_·6H_2_O and 0.1 mL of 5% (w/v) NaNO_2_ and 0.3 mL of 1 M NaOH were orderly added into a new tube and were fully mixed. The absorbance was recorded at 510 nm using an UV-visible spectrophotometer. The TFC was calculated based on a standard curve of catechin and the results were expressed as milligram catechin equation per kilogram of fresh weight (mg kg^-1^ FW).

### Determination of hydrogen peroxide (H_2_O_2_), superoxide radicals (*O*_2_·−) and malondialdehyde (MDA) contents

The H_2_O_2_ and MDA and *O*_2_·− contents were respectively estimated using commercial kits (D799773, D799761 and D799771, Sangong Biotech, China) according to the manufacturer’s instructions. The results of *O*_2_·−, H_2_O_2_ and MDA contents based on fresh weight (FW) were represented as mmol kg^-1^, μmol kg^-1^, and mmol kg^-1^, respectively.

### Assay of the activity of antioxidant enzymes in peppermint

The activities of superoxide dismutase (SOD), catalase (CAT), peroxidase (POD), and polyphenol oxidase (PPO) were detected by the method described previously (Wang and Zhu, 2017; Wang et al., 2022). First, crude enzyme was prepared before activity measurement. The 1.0 g of fresh leaf samples was homogenized with a 5 ml of 1 mM phosphate buffer (pH 7.0) containing 2% of polyvinylpyrrolidone under 4 °C ice bath. The homogenates were centrifuged at 12, 000× g for 15 min at 4 °C. The supernatants were collected to determine the activity of related enzymes.

One unit of SOD activity was defined as the quantity of enzyme which causes a 50% inhibition of nitro blue tetrazolium reduction at 560 nm. One unit of CAT activity was defined as the quantity of enzyme that decomposes 1 μmol of H_2_O_2_ per min at 240 nm. One unit of POD activity was defined as the quantity of enzyme that oxidizes 20 mM concentrated guaiacol at 470 nm. The PPO activity was measured by recording the decline of 4-methylcatechol at 398 nm. The unit of U kg^-1^ on a fresh weight basis was employed for expressing the activities of the above enzymes.

### Statistical analysis

There were three biological replicates for each experiment. The Statistical Package for Social Science (SPSS) (SPSS Inc.) 20.0 software was employed to conduct statistical analysis. The results of all measurements were presented as the mean ± standard error (*SE*). Analysis of statistical differences was performed using one-way analysis of variance (ANOVA) or student’s t-test in the SPSS. P values less than 0.05 were considered to represent significance.

## Results

### The growth performance of peppermint young plants under different concentrations of Cd treatments

**Figure 1:**
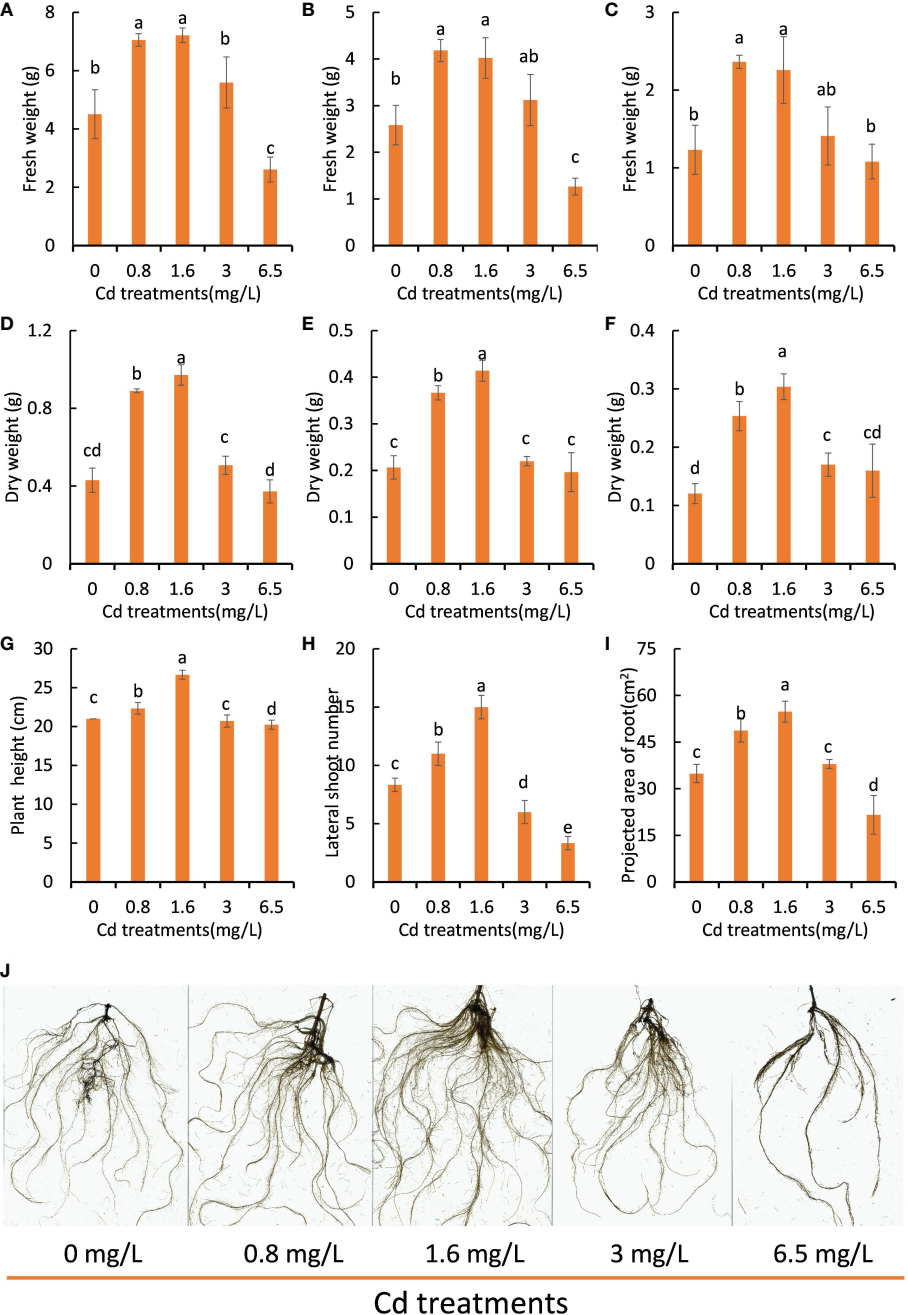
The growth performance of peppermint young plants under different concentrations of Cd treatments. **(A–C)**, fresh weight of leaves and shoots and roots, respectively; **(D–F)**, dry weight of leaves and shoots and roots, respectively; **(G)**, the height of young plants; **(H)**, the number of lateral shoots; **(I)**, projected area of root; **(J)**, root morphology. The growth performance of young plants was determined following 7 days of Cd stress treatments. Each value is presented as means ± standard error from three repeats. Statistical differences (p ≤ 0.05) between treatments are compared in the SPSS software and indicated using different letters above the bars.

As it is difficult to precisely control the concentration of Cd ion in the Cd-polluted soils, Cd stress treatment experiments were conducted with Hoagland cultivation containing different concentrations of Cd ion to investigate the effects of different degrees of Cd stress on the growth of peppermint young plants. Under Cd stress treatment for 7 days, the biomass (fresh and dry weight) of peppermint young plants first increased and then decreased with the elevation of the concentration of Cd ion and reached the highest amount at a Cd concentration of 1.6 mg L^-1^ (**Figures 1A–F**). Moreover, the cutting height (**Figure 1G**) and number of lateral branch (**Figure 1H**) and projected area of root (**Figure 1I**) in low levels of Cd treatments (0.8 and 1.6 mg L^-1^) were significantly higher than that in other treatments. In addition, low levels of Cd treatments (0.8 and 1.6 mg L^-1^) obviously promoted the development of root system (**Figure 1J**). The root morphology of the 0 mg L^-1^ and 3.0 mg L^-1^ Cd groups was also similar in appearance.

Compared with the control (0 mg L^-1^), the visible biomass decrease and root inhibition were not observed until the Cd concentration reached 3.0 mg L^-1^, suggesting that peppermint young plants exhibited strong Cd resistance. 6.5 mg L^-1^ Cd treatment retarded the growth of peppermint young plants (**Figures 1A–I**) and the roots tended to be dark, suggesting that this Cd dosage would cause toxicities for peppermint (**Figure 1J**), and thus resulting in a reduced biomass.

In order to test whether the effects of Cd treatments on peppermint growth performance is hermetic, the data on fresh weight of the whole plant were adjusted to the Brain and Cousens model (Lushchak, 2014; Guzmán-Báez et al., 2021). An inverted U-shaped dose-response hormetic curve was noted, which was characterized by stimulation at low Cd concentrations but inhibition at high Cd concentrations (**Supplementary Figure 2**). The fresh weight of the whole plant had a maximum response at Cd concentration between 0.8 and 1.6 mg/L. The mean value of this variable decreased when the Cd concentration exceeded 3 mg/L. These results together strongly demonstrated that Cd treatment triggered hormesis on peppermint growth. However, the potential mechanisms of the Cd-induced hormesis have not been revealed to date in peppermint.

### Global features and quality evaluation of RNA-seq data

Transcriptomic analysis of peppermint leaves in response to low and high degrees of Cd stress would shed light on the underlying mechanisms of the Cd-induced hormesis effects. Cd treatment at 1.6 mg L^-1^ concentration showed the best promoting effects on young plant growth (the highest biomass), but Cd treatment at 6.5 mg L^-1^ concentration significantly resulted in retrograde effects on the growth performance (**Figure 1**). Therefore, we analyzed the transcriptome profiles of young plants growing under low level of Cd (1.6 mg L^-1^), high level of Cd (6.5 mg L^-1^), and control condition (0 mg L^-1^ Cd) in this study, in order to reveal possible mechanisms of hormesis that was induced by low-level Cd exposure.

A total of 21 cDNA libraries involving 7 treatment groups (control at 0 h, 24 h and 72 h, 1.6 mg L^-1^ Cd treatment at 24 h and 72 h, 6.5 mg L^-1^ Cd treatment at 24 h and 72 h, respectively) that each group included three biological replicates were constructed. The constructed libraries were then sequenced through Illumina HiSeq platform. In total, 132.49 Gb of clean data were generated, and the size of clean data for each sample exceeded 5.70 Gb. The GC contents of the 21 libraries ranged from 48.89% to 50.17% with a mean value of 49.52%, and the minimum of Q30 percentage was 92.73% (**Supplementary Table 2**). After assembly, a total of 97,329 unigenes were generated, of which the lengths of 20,113 unigenes were longer than 1 Kb. The N50 length of unigenes was 1,404 bp and the mean length of unigenes was 734.16 bp (**Supplementary Table 3**). All unigenes were aligned against several public databases including NCBI Nr, Pfam, Swiss-Prot, KOG, KEGG, GO and COG databases (**Supplementary Data 1**), and the successful annotated percentage was 46.60% (**Supplementary Table 4**). The mapping efficiency of 21 samples based on the blast varied from 67.52% to 69.42%, as shown in **Supplementary Table 5**. These results indicated that the quality of transcriptome data was high and met the needs for further bioinformatics analysis.

### Transcriptome analysis of peppermint leaves in response to different levels of Cd stress treatment for 24 h

The transcriptomic profiles of peppermint leaf in the (control) (0 mg/L) and low-level (1.6 mg/L) and high-level (6.5 mg/L) Cd treatments were obtained when young plants were treated for 24 h. The expression differences of genes were compared within three treatments using pairwise comparison, and the differentially expressed genes (DEGs) were divided into up- or down-regulated transcripts.

**Figure 2:**
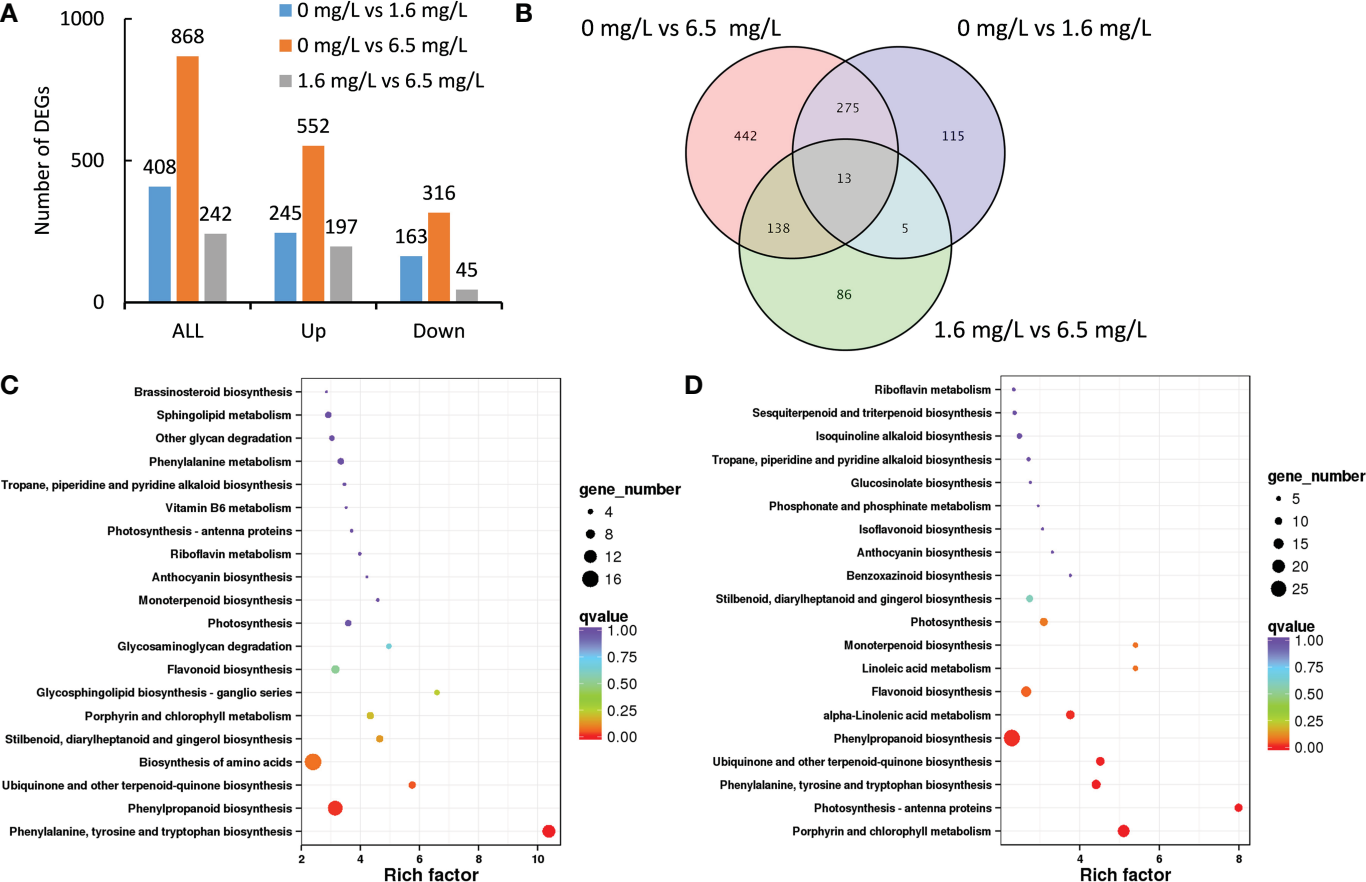
Transcriptomic profiles of peppermint leaf in response to low (1.6 mg L^-1^) and high (6.5 mg L^-1^) levels of Cd stress treatments at 24 h. **(A)**, the number of up- or down-regulated differentially expressed genes (DEGs); **(B)**, the venn diagram of DEGs; **(C)**, KEGG enrichment pathway of DEGs from the comparison of 0 mg L^-1^ Cd treatment vs 1.6 mg L^-1^ Cd treatment; **(D)**, KEGG enrichment pathway of DEGs from the comparison of 0 mg L^-1^ Cd treatment vs 6.5 mg L^-1^ Cd treatment.

A total of 408 DEGs were identified between the comparison of control and low-level Cd treatment (**Figure 2A**). Among them, low-level Cd treatment induced the expression of 245 DEGs, but repressed the expression of 163 DEGs. A total of 868 DEGs were identified between the comparison of control and high-level Cd treatment (**Figure 2A**). In which, the expression of 552 DEGs were up-regulated but that of 316 DEGs were down-regulated by high-level Cd treatment. There were 242 DEGs in the comparison group of low- and high-level Cd treatment, and most of them were up-regulated by high-level Cd stress (**Figure 2A**). This result implies that high level of Cd treatment induced more extensive responses to Cd stress at the initial stage.

Compared with the control, the expression of 580 DEGs (442 plus 138) were specifically affected by high level of Cd stress treatment, and the expression of 120 DEGs (115 plus 5), by low level of Cd treatment (**Figure 2B**). A total of 13 DEGs were commonly detected in all three comparisons. 288 DEGs (275 plus 13) were commonly identified in the comparison groups of 0 mg L^-1^ vs 6.5 mg L^-1^ and 0 mg L^-1^ vs 1.6 mg L^-1^, suggesting these DEGs might involve in Cd stress response. 151 DEGs (138 plus 13) were commonly found in the comparison groups of 0 mg L^-1^ vs 6.5 mg L^-1^ and 1.6 mg L^-1^ vs 6.5 mg L^-1^. Only 18 DEGs (5 plus 13) were commonly screened in the comparison groups of 0 mg L^-1^ vs 1.6 mg L^-1^ and 1.6 mg L^-1^ vs 6.5 mg L^-1^ (**Figure 2B**).

KEGG enrichment analysis was employed to predict the biological functions of those DEGs. The functional annotation of DEGs would provide useful information for understanding the mechanisms how peppermint leaves respond to different levels of Cd stress. The 20 most enriched KEGG pathways were displayed in **Figures 2C, D**.

In the comparison group of 0 mg L^-1^ vs 1.6 mg L^-1^, DEGs were significantly enriched in four pathways, including biosynthesis of amino acids, ubiquinone and other terpenoid-quinone biosynthesis, phenylpropanoid biosynthesis and phenylalanine, tyrosine and tryptophan biosynthesis (**Figure 2C**). The number of DEGs in three pathways including biosynthesis of amino acids (16 genes), phenylpropanoid biosynthesis (14 genes), and phenylalanine, tyrosine and tryptophan biosynthesis (12 genes) were more than 10 genes.

In the comparison group of 0 mg L^-1^ vs 6.5 mg L^-1^, porphyrin and chlorophyll metabolism, and photosynthesis-antenna proteins were the two most enriched pathways, in which 18 and 11 DEGs were identified, respectively (**Figure 2D**). These results imply that high level of Cd stress might cause damages on the photosynthesis system of peppermint leaf, despite of undergoing short-term (24 h) Cd exposure. In addition, three pathways, phenylalanine and tyrosine and tryptophan biosynthesis, ubiquinone and other terpenoid-quinone biosynthesis, and phenylpropanoid biosynthesis, were also significantly enriched. Most DEGs were enriched in the pathway of phenylpropanoid biosynthesis (26 genes) (**Figure 2D**).

Based on the results obtained, two levels of Cd stress treatments might influence the phenylpropanoid biosynthesis, and the increased phenylpropanoid content would provide protective effects on peppermint under Cd stress condition to reduce Cd- triggered oxidative damages.

### Transcriptome analysis of peppermint leaves in response to different levels of Cd stress treatment for 72 h

**Figure 3:**
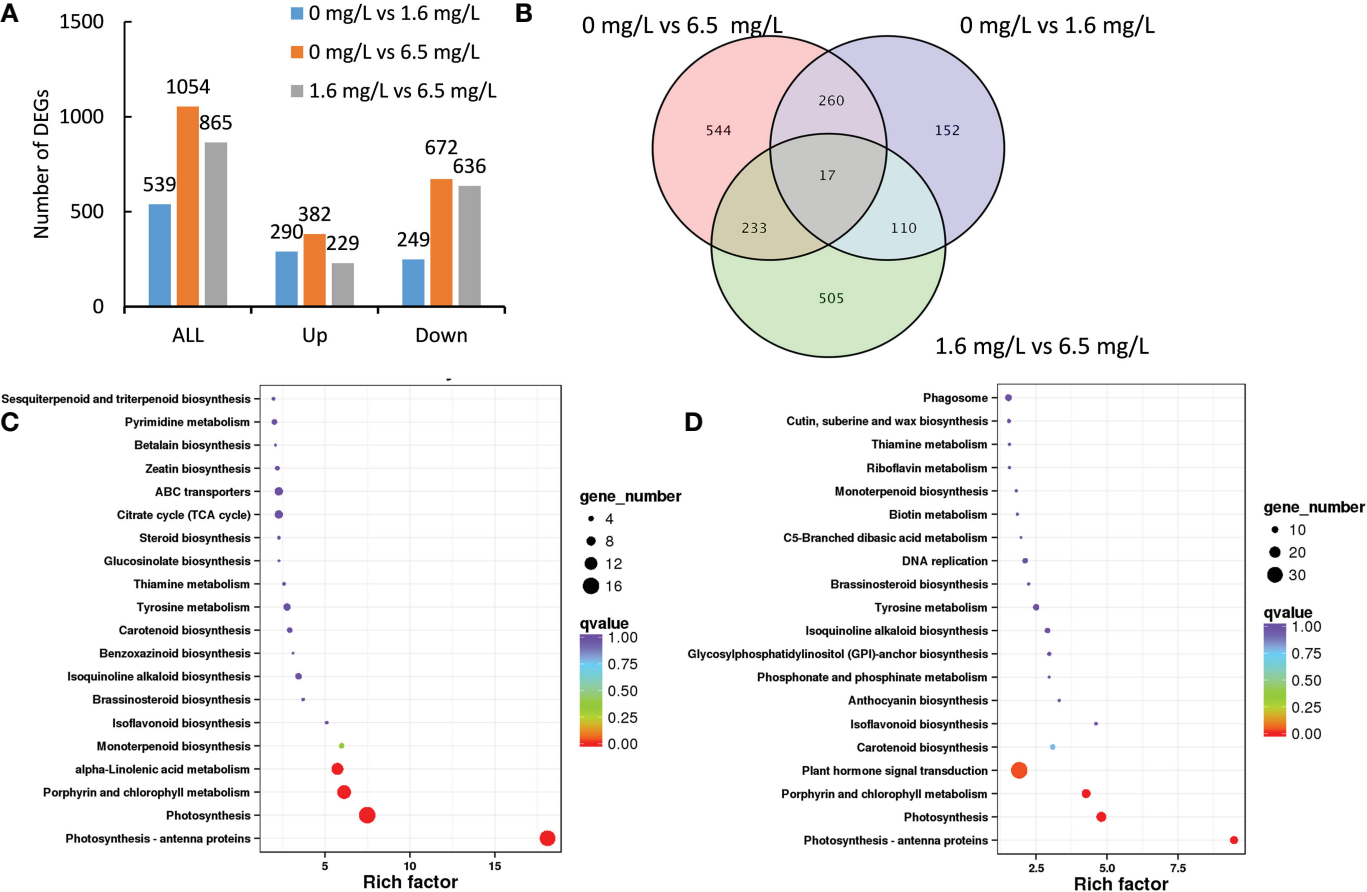
Transcriptomic profiles of peppermint leaf in response to low (1.6 mg L^-1^) and high (6.5 mg L^-1^) levels of Cd stress treatments at 72 h. **(A)**, the number of up- or down-regulated differentially expressed genes (DEGs); **(B)**, the venn diagram of DEGs; **(C)**, KEGG enrichment pathway of DEGs from the comparison of 0 mg L^-1^ Cd treatment vs 1.6 mg L^-1^ Cd treatment; **(D)**, KEGG enrichment pathway of DEGs from the comparison of 0 mg L^-1^ Cd treatment vs 6.5 mg L^-1^ Cd treatment.

A total of 539 DEGs were identified from the comparison between the control and 1.6 mgL^-1^ Cd treatment, of these DEGs, 290 DEGs were up-regulated and 249 DEGs were down-regulated by low-level Cd treatment (**Figure 3A**). High-level Cd treatment (6.5 mgL^-1^) significantly affected the expression of 1054 DEGs comparing the control, in which, 382 DEGs were induced but 672 DEGs were repressed by high-level Cd treatment (**Figure 3A**). The number of down-regulated DEGs were far more than that of up-regulated DEGs in high-level Cd treatment (**Figure 3A**). Additionally, there were 865 DEGs in the comparison group of low- and high-level Cd treatment, and most of them (636 genes) were down-regulated by high-level Cd stress treatment (**Figure 3A**). These results suggest that long-term (72 h) Cd heavy stress might repress stress responses or heavy Cd stress had caused deadly damages on peppermint leaves, and young plants were no more “vigour” to reply to Cd stress.

Compared with the control, the expression of 787 DEGs (554 plus 233) were specifically affected by 6.5 mg L^-1^ Cd treatment, and the expression of 262 DEGs (152 plus 110), by 1.6 mg L^-1^ Cd treatment (**Figure 3B**). It was found that 17 DEGs were commonly detected in all three comparisons. 277 DEGs (260 plus 17) were commonly identified in the comparison groups of 0 mg L^-1^ vs 6.5 mg L^-1^ and 0 mg L^-1^ vs 1.6 mg L^-1^. 250 DEGs (233 plus 17) were commonly detected in the comparison groups of 0 mg L^-1^ vs 6.5 mg L^-1^ and 1.6 mg L^-1^ vs 6.5 mg L^-1^. (**Figure 3B**). 127 DEGs (110 plus 17) were commonly discerned in the comparison groups of 0 mg L^-1^ vs 1.6 mg L^-1^ and 1.6 mg L^-1^ vs 6.5 mg L^-1^ and 110 DEGs were overlapped in these two comparisons (**Figure 3B**).

In the comparison group of 0 mg L^-1^ vs 1.6 mg L^-1^, DEGs were significantly enriched in the pathways of photosynthesis (16 genes), photosynthesis-antenna proteins (15 genes), porphyrin and chlorophyll metabolism (13 genes) and alpha-Linolenic acid metabolism (11 genes) (**Figure 3C**). In the comparison group of 0 mg L^-1^ vs 6.5 mg L^-1^, DEGs were significantly enriched in the pathways of plant hormone signal transduction (33 genes), photosynthesis (17 genes), porphyrin and chlorophyll metabolism (15 genes), and hotosynthesis-antenna proteins (13 genes) (**Figure 3D**). These results together suggest that long-term Cd stress might injure photosynthetic system of the peppermint leaves, but the mechanisms regarding hormesis effects induced by low-level Cd exposure were unclear.

The most DEGs (33 genes) were enriched in the pathway of plant hormone signal transduction (**Figure 3D**), suggesting that endogenous hormones were involved in the Cd stress response under high-level Cd condition.

### Functional annotation of DEGs exclusively induced by low level of Cd exposure

**Figure 4:**
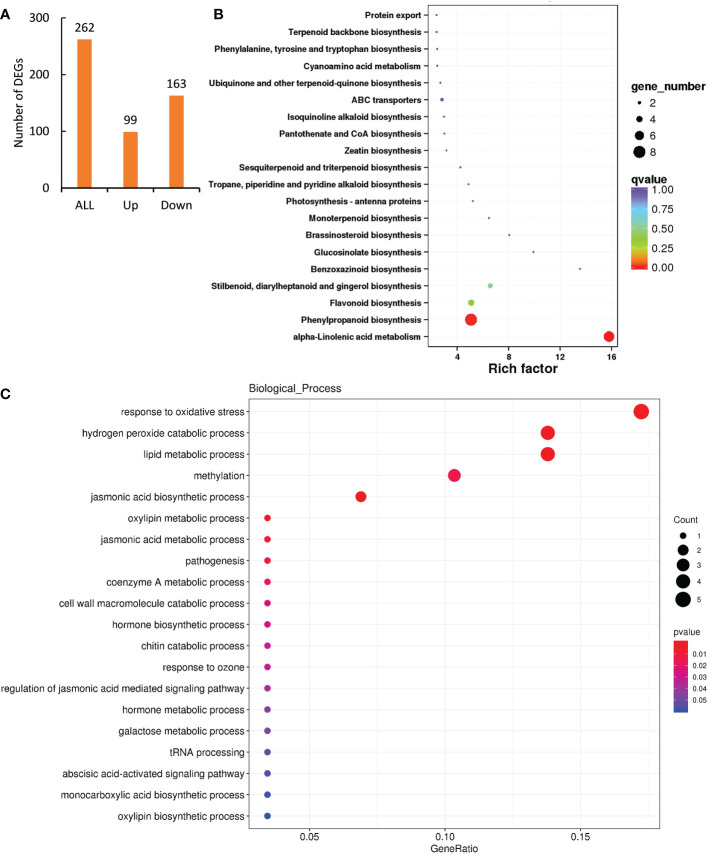
Functional annotation of DEGs exclusively induced by low level (1.6 mg L^-1^) of Cd exposure for 72 h. **(A)**, the number of DEGs exclusively affected by 1.6 mg L^-1^ Cd exposure at 72 h; **(B)** GO and **(C)** KEGG enrichment analysis of DEGs exclusively induced by 1.6 mg L^-1^ Cd treatment.

As low level of Cd treatment induced significant hormesis effects on peppermint growth (**Figure 1**), analyzing the functional roles of DEGs exclusively induced by 1.6 mg L^-1^ Cd treatment would reveal the hormesis effects mediated by that. A total of 262 DEGs were exclusively affected by low level of Cd exposure (**Figure 4A**). Among them, the expressions of 99 DEGs were significantly up-regulated by 1.6 mg L^-1^ Cd treatment comparing with both 0 and 6.5 mg L^-1^ Cd treatments (**Figure 4A**), signifying the important roles of them in Cd-induced hormesis in peppermint young plants. Therefore, we mainly analyzed the functional roles of the induced 99 DEGs through GO and KEGG enrichment analysis.

KEGG enrichment analysis showed that the two most enriched pathways were phenylpropanoid biosynthesis and alpha-Linolenic acid metabolism, which comprised of 8 and 7 DEGs, respectively (**Figure 4B**). 4 DEGs were also enriched in the pathway of flavonoid biosynthesis.

GO analysis showed that the induced DEGs were mainly enriched in the terms of response to oxidative stress, hydrogen peroxide catabolic process and lipid metabolic process in the biological process category (**Figure 4C**), integral component of membrane in cellular component category, and heme binding, oxidoreductase activity, methyltransferase activity, iron ion binding and peroxidase activity in the category of molecular function (**Supplementary Figure 3**).

Apart from antioxidant enzyme, many phenylpropanoids and flavonoids are known non-enzymatic antioxidants (Zhou et al., 2022). And therefore, the results of GO and KEGG analysis pointed to antioxidant system. We supposed that low-level Cd exposure might induce strong antioxidant activity under mild Cd stress condition, and thus avoiding oxidative damages for peppermint tissues and manifesting as stimulative effects on cutting growth. Therefore, the analysis was focused on the biosynthesis phenylpropanoid and flavonoid and enzymatic antioxidant activities of peppermint young plants under different degrees of Cd stress conditions.

### Effects of different degrees of Cd stress treatments on the biosynthesis of phenylpropanoids and flavonoids and quinones

**Figure 5:**
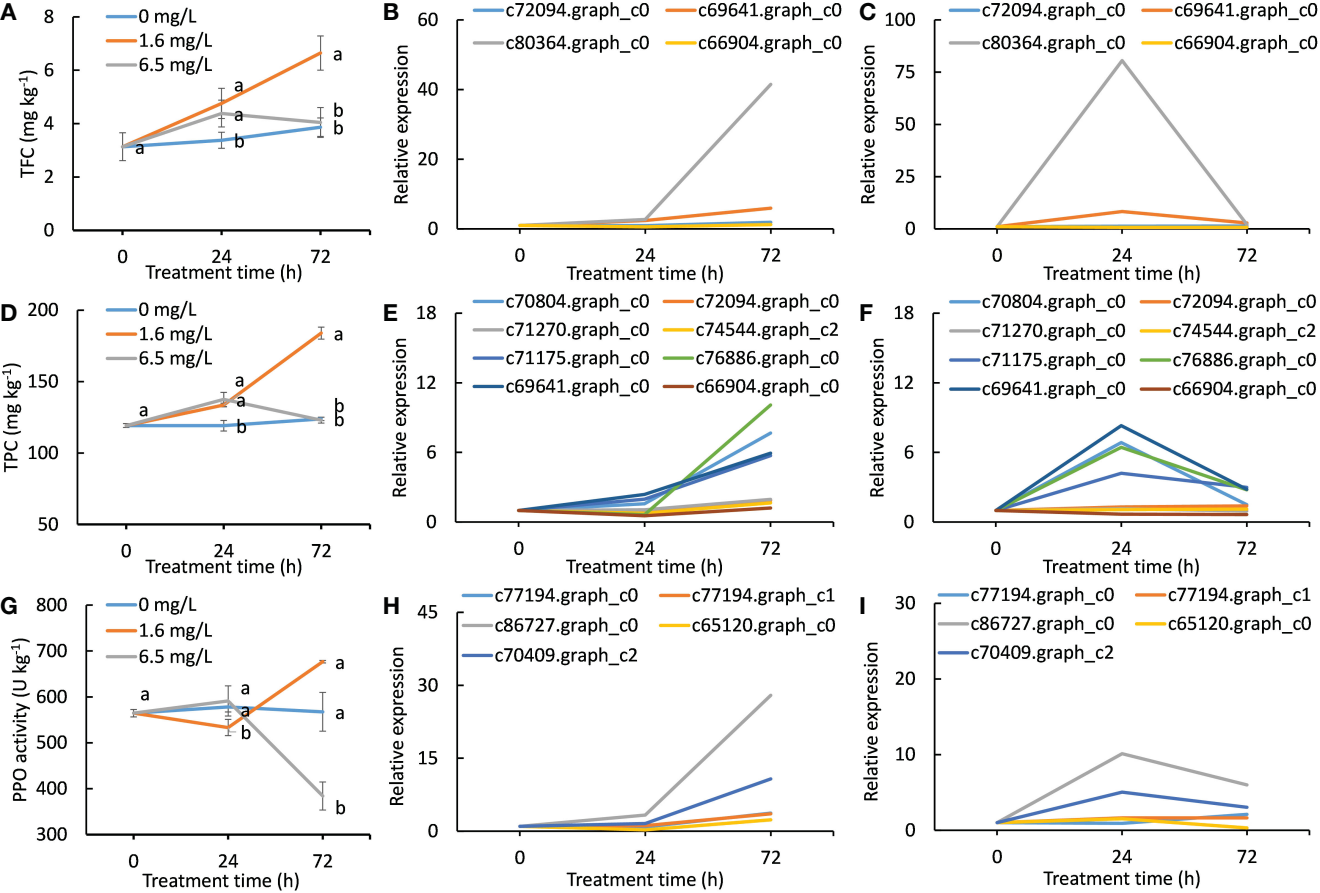
Effects of different degrees of Cd stress treatments on the biosynthesis of phenylpropanoids and flavonoids and quinones. **(A)**, total flavonoid contents of different Cd treatments in peppermint leaves; **(B, C)**, the expression patterns of DEGs involved in the biosynthesis of flavonoids during low level of Cd treatment (1.6 mg L^-1^) and high level of Cd treatment (6.5 mg L^-1^), respectively; **(D)**, total phenolic contents in peppermint leaves; **(E, F)**, the expression patterns of DEGs involved in the biosynthesis of phenylpropanoid during low level of Cd treatment (1.6 mg L^-1^) and high level of Cd treatment (6.5 mg L^-1^), respectively; **(G)**, polyphenol oxidase (PPO) activity; **(H, I)**, the expression patterns of DEGs encoding PPO during low level of Cd treatment (1.6 mg L^-1^) and high level of Cd treatment (6.5 mg L^-1^), respectively. Each value is presented as means from three repeats. In figures **(A, D, G)**, statistical differences (p ≤ 0.05) between treatments are compared in the SPSS software and indicated using different letters above the bars.

Total flavonoid contents (TFCs) of the control were not obviously increased during the entire period of 72 h cultivation. The TFCs of two Cd treatments were significantly increased after 24 h Cd treatment when compared with the control (**Figure 5A**). However, the increased TFCs in 6.5 mg L^-1^ Cd treatment was then declined to the same level as the control at 72 h. Therefore, an inverted V-shape was formed during high-level Cd exposure. But TFCs in 1.6 mg L^-1^ Cd treatment was constantly increased and which showed significantly higher levels when compared with the control and 6.5 mg L^-1^ Cd treatment (**Figure 5A**).

A total of 4 DEGs involving in the flavonoid biosynthesis were identified. The expression levels of all DEGs were continuously up-regulated during low-level Cd exposure (**Figure 5B**), while their expression levels were reached to peaks at 24 h and then declined at 72 h in high-level Cd stress treatment (**Figure 5C**). The overall expression pattern of 4 DEGs in flavonoid biosynthesis was highly consistent with the change trends of TFCs during Cd treatment (**Figures 5A–C**). One gene (c80364.graph_c0) was strongly responded to Cd treatments, implying that this gene might play a dominant role in the biosynthesis of flavonoid compounds under Cd stress condition.

The change trends of total phenolic content (TPC) in three treatments were similar with TFC during Cd stress treatment (**Figure 5D**). The TPCs in low level of Cd treatment were continually increased as the time goes in the course of the Cd treatment, and that was significantly higher than that in the control at any time point, whereas that in high level of Cd treatment only showed significantly higher levels than the control at 24 h (**Figure 5D**). Similarly, an inverted V-shape for TPC was formed during high-level Cd exposure.

A total of 8 DEGs were identified that they were involved in the phenylpropanoid biosynthesis. In low level of Cd treatment, the expression levels of all 8 DEGs were increased with the evaluation of treatment time and the expression levels of four genes (c76886.graph_c0, c70804.graph_c0, c71175.graph_c0 and c69641.graph_c0) were sharply increased after 24 h (**Figure 5E**). However, the expression of these four genes were merely induced by high level of Cd stress treatment at 24 h but that were reduced thereafter. The rest of 4 genes (c71270.graph_c0, c72094.graph_c0, c74544.graph_c2 and c66904.graph_c0) were not significantly induced by high-level Cd treatment at any time point (**Figure 5F**).

PPO is a key enzyme in the formation of quinone compounds in plant (Sullivan, 2015). The PPO activity of the control had barely risen at all during 72 h. In low level of Cd treatment, PPO activity first slightly reduced at 24 h and then sharply increased at 72 h. PPO activity of high level of Cd treatment showed an opposite trend with that of low level of Cd treatment (**Figure 5G**). At 72 h, PPO activity of low level of Cd treatment was significantly higher than that of both the control and high level of Cd treatment, and that of high level of Cd treatment was significantly lower than that of the control (**Figure 5G**). There were 5 DEGs encoding PPO. The expression levels of all *PPO* genes were continuously increased during Cd treatment in low level of Cd treatment (**Figure 5H**), while that were just induced by high-level Cd treatment at 24 h (**Figure 5I**). These results together demonstrate that Cd stress treatment induced the formation of quinones, and the biosynthesis ability of quinones was inhibited after long exposure to high-level Cd stress in peppermint leaves.

For the TFC, TPC, PPO activity and the expression patterns of genes in the biosynthesis of phenylpropanoids and flavonoids and quinones, inverted V-shapes were formed during high-level Cd exposure, suggesting that the induction of the biosynthesis of those antioxidants under Cd stress might be important mechanisms to adapt Cd stress, and high dosage of Cd stress just induced a temporary action on their biosynthesis and thus resulting in failure to cope with a sustained heavy Cd stress. However, low-level Cd exposure constantly induced their biosynthesis and accumulation, which would provide sufficient antioxidants to alleviate Cd toxicity. The genes responsible for the biosynthesis of phenylpropanoids and flavonoids and quinones were not activated by Cd stress at the same time, they were induced by an order manner under a yet unknown mechanism.

### Effects of different degrees of Cd stress treatments on enzymatic antioxidant activity

**Figure 6:**
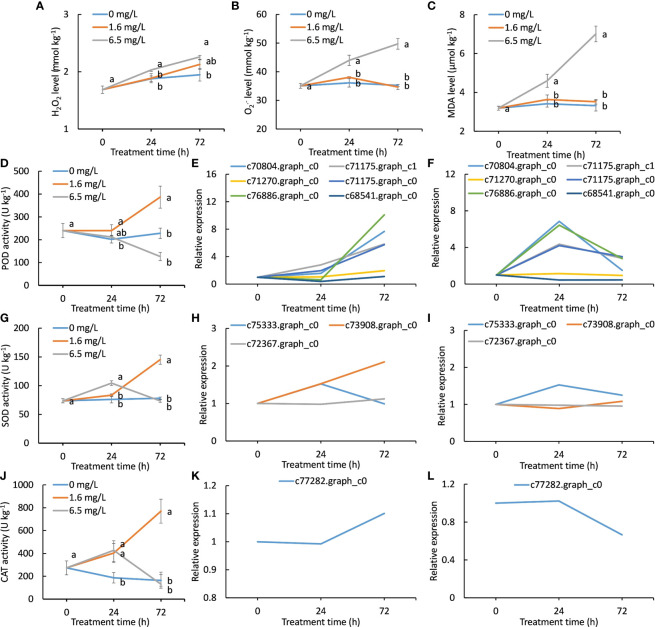
Changes of antioxidant activity and the expression patterns of DEGs encoding antioxidant enzymes during Cd exposure. **(A)**, hydrogen peroxide (H_2_O_2_) contents of different Cd treatments in peppermint leaves; **(B)**, superoxide anion (*O*_2_·−) contents; **(C)**, malondialdehyde (MDA) contents; **(D)**, peroxidase (POD) activity; **(E, F)**, the expression patterns of DEGs encoding POD during low level of Cd treatment (1.6 mg L^-1^) and high level of Cd treatment (6.5 mg L^-1^), respectively; **(G)**, superoxide dismutase (SOD) activity; **(H, I)**, the expression patterns of DEGs encoding SOD during low level of Cd treatment (1.6 mg L^-1^) and high level of Cd treatment (6.5 mg L^-1^), respectively; **(J)**, catalase (CAT) activity; **(K, L)**, the expression patterns of DEG encoding CAT during low level of Cd treatment (1.6 mg L^-1^) and high level of Cd treatment (6.5 mg L^-1^), respectively. Each value is presented as means from three repeats. In figures **(A-D, G, J)**, statistical differences (p ≤ 0.05) between treatments are compared in the SPSS software and indicated using different letters above the bars.

The change trends of H_2_O_2_, *O*_2_·− and MDA concentrations were similar during Cd treatment (**Figures 6A–C**). The 6.5 mg L^-1^ Cd treatment progressively increased in contents of H_2_O_2_, ${\text{O}}_{2}^{\mathrm{\cdot -}}$ and MDA with the extended treatment, whereas those of the 0 and 1.6 mg L^-1^ Cd treatments increased much less, indicating that heavy Cd stress triggered oxidative burst. More importantly, no significant differences in contents of H_2_O_2_, *O*_2_·− and MDA between the control and low-level Cd treatment were observed (**Figures 6A–C**), suggesting that low-level Cd exposure did not bring about severe oxidative damages on peppermint young plants. This might be one of the reasons why peppermint young plants could well grow under low levels of Cd treatment but not, under high degree of Cd stress.

POD activity of the control was changed much less during 72 h cultivation. POD activity of high-level Cd treatment was reduced with treatment time, suggesting that high-level Cd treatment inactivated POD activity *via* a yet unknown mechanism. POD activity of low-level Cd treatment was sharply increased after 24 h and was significantly higher than that of the other two treatments at 72 h (**Figure 6D**). Six DEGs encoding POD were exclusively induced by low-level Cd treatment. The expression levels of all genes were increased with treatment time in low-level Cd treatment (**Figure 6E**). In high-level Cd treatment, the expression levels of four genes (c70804.graph_c0, c76886.graph_c0, c71175.graph_c0 and c71175.graph_c1) were first increased at 24 h and reduced thereafter (**Figure 6F**). Two genes (c71270.graph_c0 and c68541.graph_c0) expression were progressively reduced as treatment time increased (**Figure 6F**).

SOD activity of the control was almost unchanged during 72 h cultivation. SOD activity of high-level Cd treatment was reached to a peak at 24 h and then reduced to low level as same as the control at 72 h. In low-level Cd treatment, SOD activity was progressively increased during Cd treatment (**Figure 6G**). Low level of Cd treatment steady induced two genes (c73908.graph_c0 and c72367.graph_c0) expression during 72 h (**Figure 6H**), while their expression was only up-regulated at 24 h by high-level Cd treatment (**Figure 6I**). The expression pattern of one gene (c75333.graph_c0) during Cd treatment was similar in low- and high-level Cd treatment.

CAT activity in the control was gradually decreased in the next 72 h cultivation. Two concentrations of Cd treatments significantly enhanced CAT activity when compared with the control at 24 h (**Figure 6J**). CAT activity of low-level Cd treatment was continued to increase but that of high level of Cd treatment were reduced after 24 h (**Figure 6J**). At 72 h, CAT activity of low-level Cd treatment was significantly higher than that of the control and of high level of Cd treatment (**Figure 6J**). Only one *CAT* gene (c77282.graph_c0) was significantly induced by low-level Cd treatment at 72 h (**Figure 6K**), while high-level Cd treatment significantly inhibited its expression (**Figure 6L**). The expression patterns in low- and high Cd treatments were diametrically opposed during Cd treatment.

### Expression verification of DEGs through qRT-PCR method

**Figure 7:**
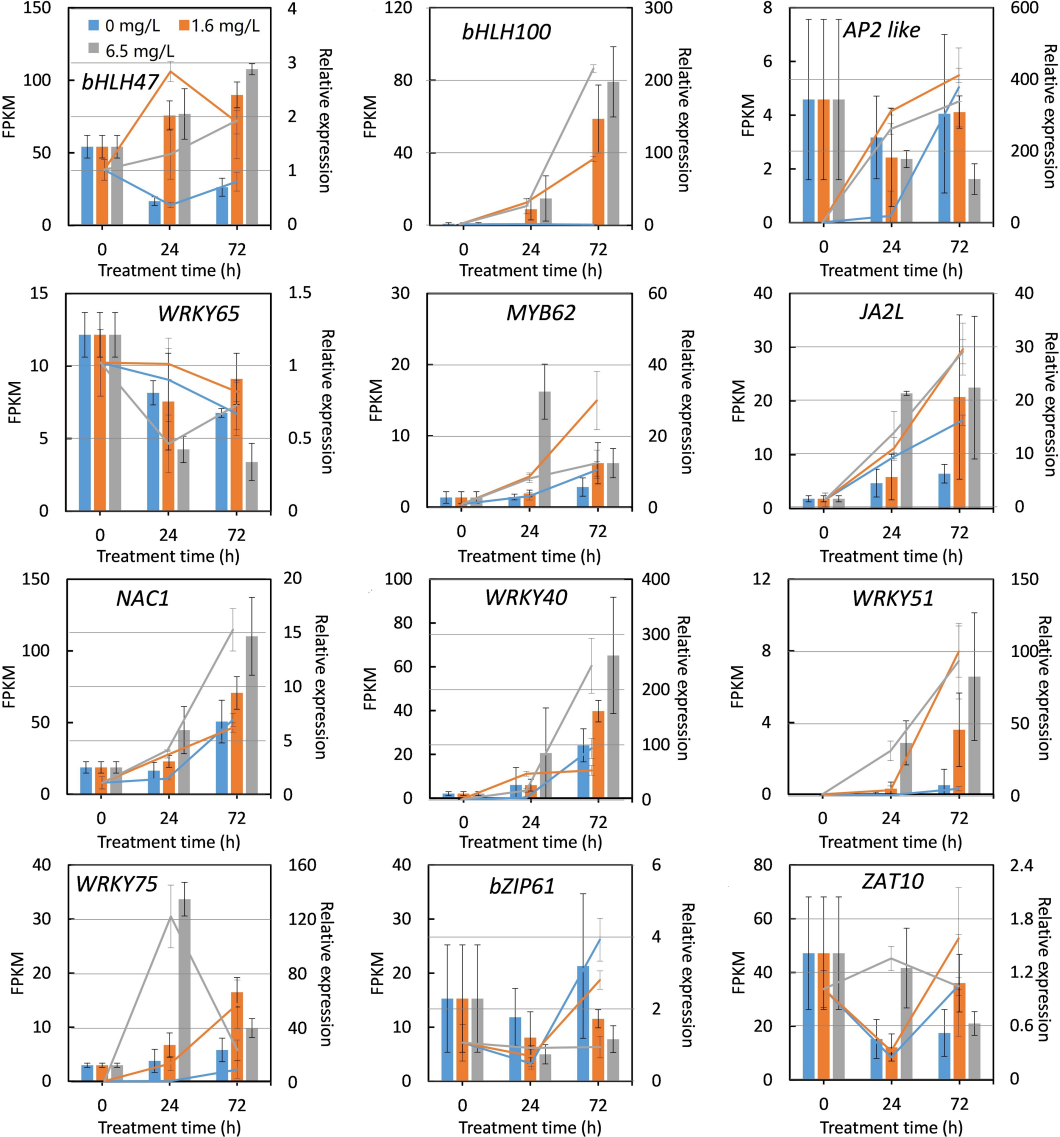
Quantitative real-time PCR (qRT-PCR) verification of the expression of 12 DEGs selected from RNA sequencing under Cd stress. The X-axis represents treatment time. The Y-axis on the left represents the expression level of specific gene obtained from RNA-seq (histogram), and on the right, from qRT-PCR analysis (line chart). Each value is presented as means ± standard error from three repeats. Standard errors are indicated in figures using the bars.

To validate the accuracy and credibility of transcriptome data, 12 DEGs were randomly selected according to the results of transcriptomic analysis to measure the relative expression levels. The expression patterns of 12 DEGs were determined at 0 h, 24 h and 72 h following different concentrations of Cd stress treatments using specific primers based on qRT-PCR method. Among 12 DEGs selected, the expression of two genes including peppermint *ZAT10* and *AP2 like* were down-regulated by Cd stress treatment, the rest of DEGs were induced in RNA-Seq data (**Figure 7**). Only one gene, *AP2 like*, displayed a converse expression pattern determined by qRT-PCR and RNA-Seq during Cd stress treatment. The overall expression patterns of the rest of 11 DEGs determined by two methods were highly consistent, although the fold changes did not match exactly (**Figure 7**). These results indicated that the RNA-seq date were reliable and the results of transcriptomic analysis were credible.

## Discussion

Peppermint traditionally grows as a cash crop in many countries (Cunningham and Ow, 1996) including China, for the production of either essential oils or herbage (Ahkami et al., 2015). In this study, we examined the effects of varying degrees of Cd stress on the growth of peppermint cuttings. The results showed that peppermint could tolerate heavy Cd stress even over 3.0 mg L^-1^ concentration (**Figure 1**). More importantly, low doses of Cd treatments (0.8 and 1.6 mg L^-1^) enhanced the growth performance of peppermint young plants. These results suggest that peppermint plant had strong tolerance to Cd stress and low-level Cd exposure would be able to increase the yield of peppermint plant. Such beneficial effects offered peppermint much opportunity for application in the safe utilization of mild Cd-contaminated farmland.

The chief purpose of peppermint is to extract essential oil (Ahkami et al., 2015). In another respect, the Cd accumulation in peppermint tissues must be given much attention to ensure the safety. To confirm the safety of peppermint oil, we also determined the contents of Cd ion in essential oil of peppermint planted on Cd-contaminated farmland soil, and found that Cd ions were not detected at all in essential oil (data not shown). The results from Youssef (2021) concur with our results, in which high concentrations of Cd treatments do not influence the essential oil quality of sweet basil. These results suggest that peppermint could act as an alternative cash crop to utilize the Cd-polluted soils safely and efficiently, which let the heavy metal-contaminated farmland soils be profitable for farmers. In addition, the most Cd were generally accumulated in roots, followed by shoots and leaves in plant (Xie et al., 2021). The increase in Cd concentration enhanced the yield and quality of essential oil in sweet basil (Youssef, 2021). These natural advantages of Cd effects help peppermint to be more suitable for safe soil utilization.

The growth promotion derived from low levels of Cd exposure in plant has recently aroused great interests from environmentalists regarding pollution control technology for recently (Xie et al., 2021). However, the poor understanding of Cd-induced hormesis mechanisms in plant restricts the application of this beneficial effect. It has been widely demonstrated that RNA seq combined with bioinformatic analysis is an effective method to reveal the molecular mechanisms of Cd tolerance in plant (Hu et al., 2014; Huang et al., 2019; Derakhshani et al., 2020). Analyzing the transcriptional changes of peppermint young plants in response to different concentrations of Cd treatments would provide useful information for understanding the Cd-induced hormesis. Thus, the transcriptional responses of peppermint leave to low- and high-level Cd ((1.6 and 6.5 mg L^-1^, respectively) were compared and analyzed at a global level, to reveal the potential mechanisms of Cd-mediated hormesis effects.

In this study, a total of 21 cDNA libraries of peppermint leaf were sequenced through Illumina HiSeq platform. After sequencing, a total of 132.49 Gb of clean data with high quality were obtained (**Supplementary Tables 2-5**), and the credibility and reliability of transcriptome data were validated through qRT-PCR method (**Figure 7**). Among the assembled unigenes, 46.60% genes were successfully annotated by public databases (**Supplementary Table 4**). The annotated rate was higher than other plant species without genomic information, i.e., 35.92% for wild paper mulberry (Xu et al., 2019) and 40.38% for *Crossostephium chinensis* (Yang et al., 2017), indicating the high quality of RNA-seq data. After comparative analysis, the expressions of many transcripts were significantly affected by different dosages of Cd treatments (**Figures 2**, **3**), suggesting that peppermint young plants made quick responses to varying Cd.

The affected DEGs by Cd treatments at initial stage (24 h) were mainly involved in the biosynthesis of amino acid and phenylpropanoid (**Figure 2**), suggesting that these biological processes were involved in the adaption to Cd stress in peppermint leaves. However, the affected DEGs at 72 h were mainly enriched in the photosynthetic system (**Figure 3**). Cd toxicity injures the photosynthetic apparatus, especially the light-harvesting complex and photosystems I and II (Hasan et al., 2019). The results imply that long-term exposure to high-level Cd brought about negative impacts on photosynthetic system in peppermint leaves. It has also been suggested that the improvement of photosynthetic efficiency by short-term exposure to Cd might be an important mechanism of hermetic effect in plant (Adamakis et al., 2020). The growth performance of peppermint young plants was improved after the application of low levels of Cd treatments (0.8 and 1.6 mg L^-1^) in this study (**Figure 1**). The results imply that low-level Cd exposure improved the photosynthetic efficiency of peppermint leaves, which might be a reason why low-level Cd treatment increased the biomass of peppermint cuttings. However, more investigations are needed to study the effects of Cd exposure on photosynthetic system in peppermint leaves.

In order to knowing more about the mechanisms of Cd-induced hermetic effects, we mainly analyzed the roles of DEGs exclusively induced by low level of Cd exposure. The results of KEGG and GO analysis showed that the DEGs were mostly enriched in the biosynthesis pathways of phenylpropanoid and flavonoid, as well as the activity of scavenging ROS (**Figure 4**). These results imply that low-level Cd exposure induced hermetic effects in peppermint cuttings, probably, by activating the antioxidant system, and the ROS scavenging ability of peppermint leaves was constantly strengthened by low-level Cd exposure. To verify our findings, we analyzed the changes of antioxidant attributes of peppermint leaves in response to different Cd treatments.

Cd stress generally stimulates the generation of ROS, which will cause oxidative damages on plant tissues (Auobi Amirabad et al., 2020). Reducing ROS accumulation or enhancing detoxification may be one of the defense mechanisms of Cd toxicity in plants (Lata et al., 2019). MDA content is an indicator of membrane liquid peroxidation (Davey et al., 2005), and the increased MDA content indicates membrane lipid peroxidation. In the present study, high-level Cd treatment resulted in significant increases in H_2_O_2_, ${\text{O}}_{2}^{\mathrm{\cdot -}}$ and MDA contents, while low-level Cd exposure inhibited their increases (**Figures 6A–C**). These results together suggest that high level of Cd treatment caused severe oxidative damages on peppermint leaves, while low level of Cd treatment did not.

A range of natural compounds act as ROS scavengers (Haider et al., 2021). These include, but are not limited to non-enzymatic scavengers, i.e., ascorbic acid, glutathione, phenylpropanoids, flavonoids, and quinones; and enzymatic scavengers; i.e., POD, CAT and SOD (Shahid et al., 2014). Phenolic acids such as some henylpropanoids and flavonoids are the key substances for Cd tolerance in plants (Gu et al., 2021). Pretreatment with proanthocyanidins significantly alleviated Cd toxicity in industrial hemp, an annual herbaceous cash crop, and proanthocyanidins treatment induced the synthesis of secondary metabolites and antioxidant compounds (Yin et al., 2022). Flavonoid amendment enhanced Cd tolerance and had a significant stimulative effect on symplasm transport of Cd in *A. marina* roots (Li et al., 2015). In this study, low-level Cd treatment constantly induced the biosynthesis of phenylpropanoids and flavonoids, and thus resulting in an elevation of total flavonoid and phenolic contents (**Figure 5**). In addition, the activities and gene expressions of PPO, a key enzyme responsive for quinone formation, were incessantly enhanced by low level of Cd treatment (**Figure 5**), suggesting that low-level Cd treatment also induced the quinone biosynthesis. These results suggest that the incessant biosynthesis of phenylpropanoids and flavonoids and quinones induced by low-level Cd treatment provided sufficient non-enzymatic antioxidants to protect cells from oxidative damages, and thus manifesting as beneficial effects on peppermint growth under Cd stress condition.

Antioxidant enzymes influence plant Cd tolerance as they play a crucial role in balancing the ROS level within the cell. Studies reported that antioxidant enzymes caused alternation in their activities, depending on Cd dosage and the cultivars of plants (Haider et al., 2021). Treatment with Cd had shown to enhance SOD activity in wheat (Milone et al., 2003). Cd toxicity trended to minimize the enzymatic activities of SOD and CAT in pea (Sandalio et al., 2001). Cd stress had no significant impact on the activity of POD in pea plant (Tran and Popova, 2013), but POD was increased in raddish treated with Cd (El-Beltagi et al., 2010). High CAT activity resulted to improve tolerance in cultivars of *Groenlandia densa* L. treated with Cd stress (Yilmaz and Parlak, 2011). In this study, we found that low level of Cd treatment significantly enhanced the activities and gene expression of POD, CAT and SOD during the entire 72 h Cd treatment (**Figure 6**). The results suggest that low-level Cd exposure could induced the activity of antioxidant enzyme at both protein and gene levels in peppermint leaves. The enhanced activity of antioxidant enzymes contributes to maintain lower ROS levels within cells.

Although high-level Cd treatment induced strong antioxidant defense response at initial stage (24 h), the induction was unsustainable and thus leading to the failure to offset Cd-triggered ROS burst. However, the antioxidant activity was continuously strengthened by low-level Cd treatment during the entire period of Cd exposure. The results strongly demonstrate that low-level Cd exposure constantly enhanced the antioxidant capacity of peppermint, which might be the possible mechanisms underlying Cd-induced hermetic effects. However, we also noticed that all the ROS scavengers were not activated by low level of Cd treatment at the same time and the induced intensity differed greatly among ROS scavengers (**Figures 5**, **6**). These results suggest that the activated mode and the induced intensity for ROS scavengers depended on Cd concentration and treatment duration. All in all, our research provided new insights for the Cd hermetic effects in peppermint young plants.

## Conclusion

Peppermint young plants showed strong Cd tolerance and low-level Cd promoted the growth performance, suggesting that planting peppermint may be a feasible strategy for the safe and efficient utilization of Cd-contaminated farmland soil. Low level of Cd exposure constantly induced the biosynthesis of phenylpropanoid and flavonoid, enhanced activities of antioxidant enzymes, while high level of Cd treatment at long period (72 h) inhibited them and caused oxidative burst, suggesting that low level of Cd exposure constantly induced the enhancement of antioxidant activity. The constant activation of antioxidant system by low-level Cd exposure suggests the possible mechanisms of hermetic effects in peppermint young plants.

## Data availability statement

The data presented in the study are deposited in the NCBI Sequence Read Archive (SRA) repository, accession number PRJNA898846. The names of the repository/repositories and accession number(s) can be found in the article/**Supplementary Material**.

## Author contributions

BW: Conceptualization, Investigation, Data analysis, Visualization, Writing-review and editing; LL: Investigation; XY: Data analysis, Writing and editing; YZ, YW, and DL: Writing and editing; JH: Project administration, Review, editing and Supervision; YX: Plant material management, Cd treatment, Writing-review and editing. All authors contributed to the article and approved the submitted version.
