# Supplementary figures and images for: Low-level cadmium exposure induced hormesis in peppermint young plant by constantly activating antioxidant activity based on physiological and transcriptomic analyses
Source: Front Plant Sci. 2023 Jan 23;14:1088285. doi: 10.3389/fpls.2023.1088285 (PMC9899930; doi:10.3389/fpls.2023.1088285)

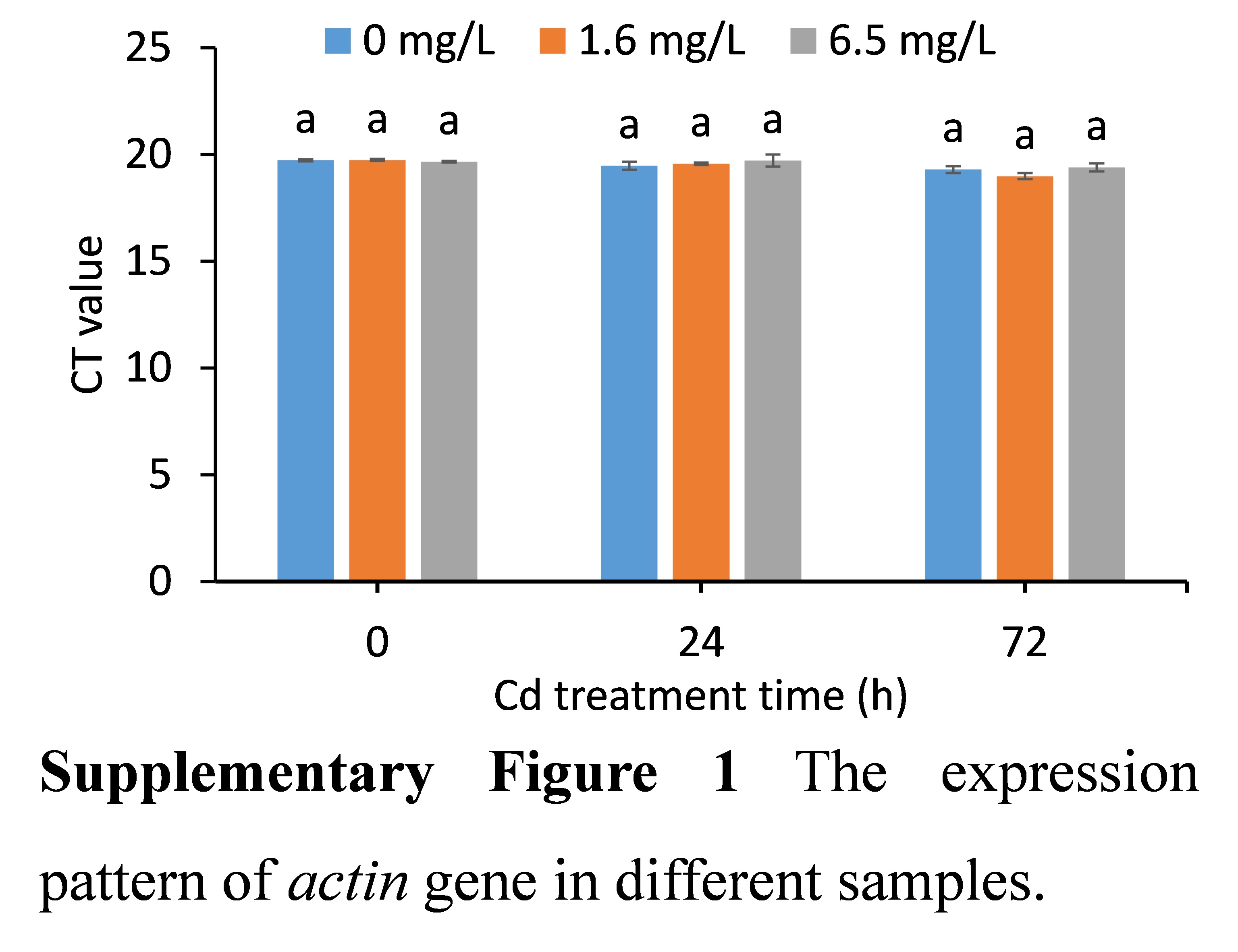

Supplement: Supplementary file 2 [file Image_1.jpeg]

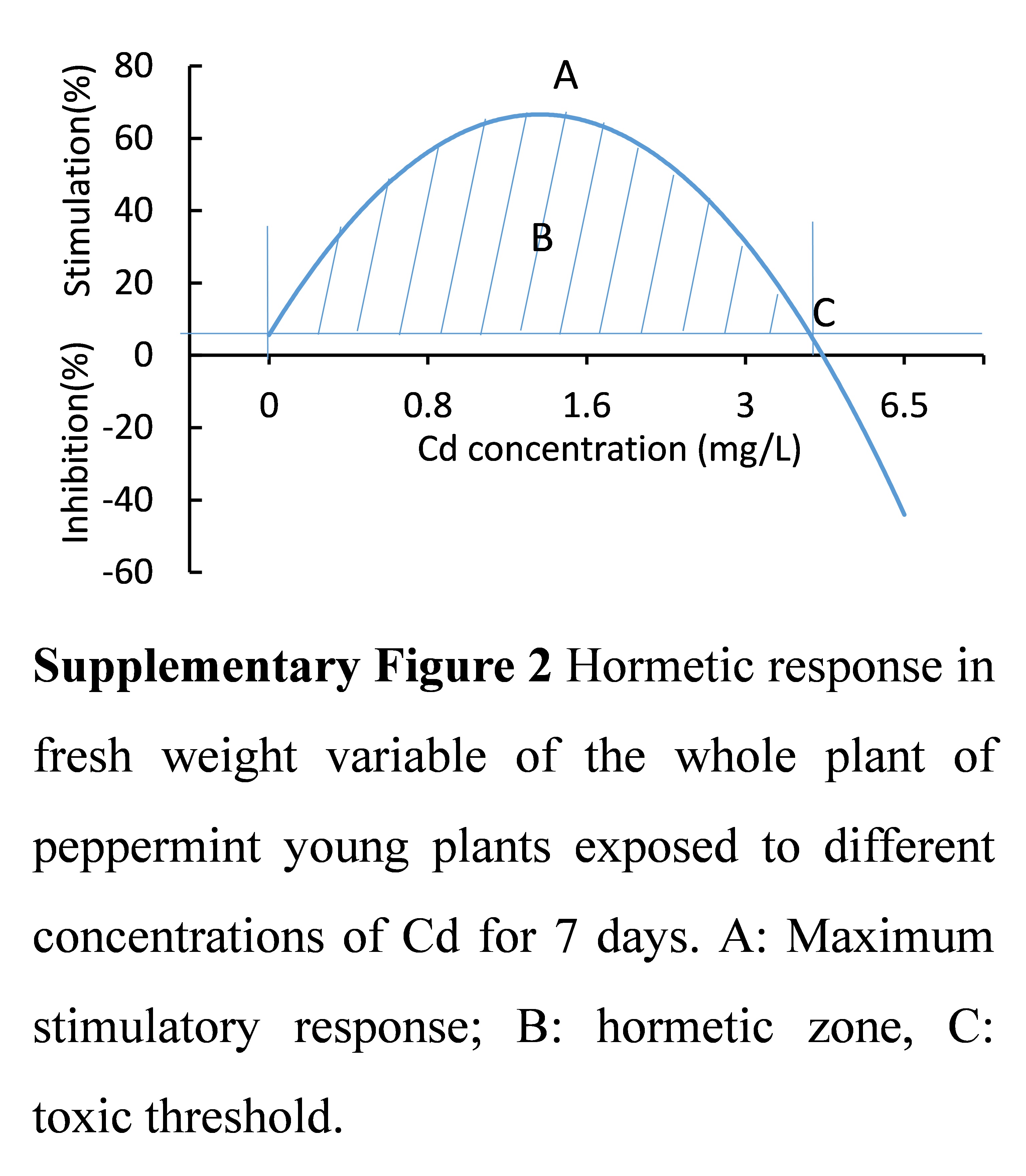

Supplement: Supplementary file 3 [file Image_2.jpeg]

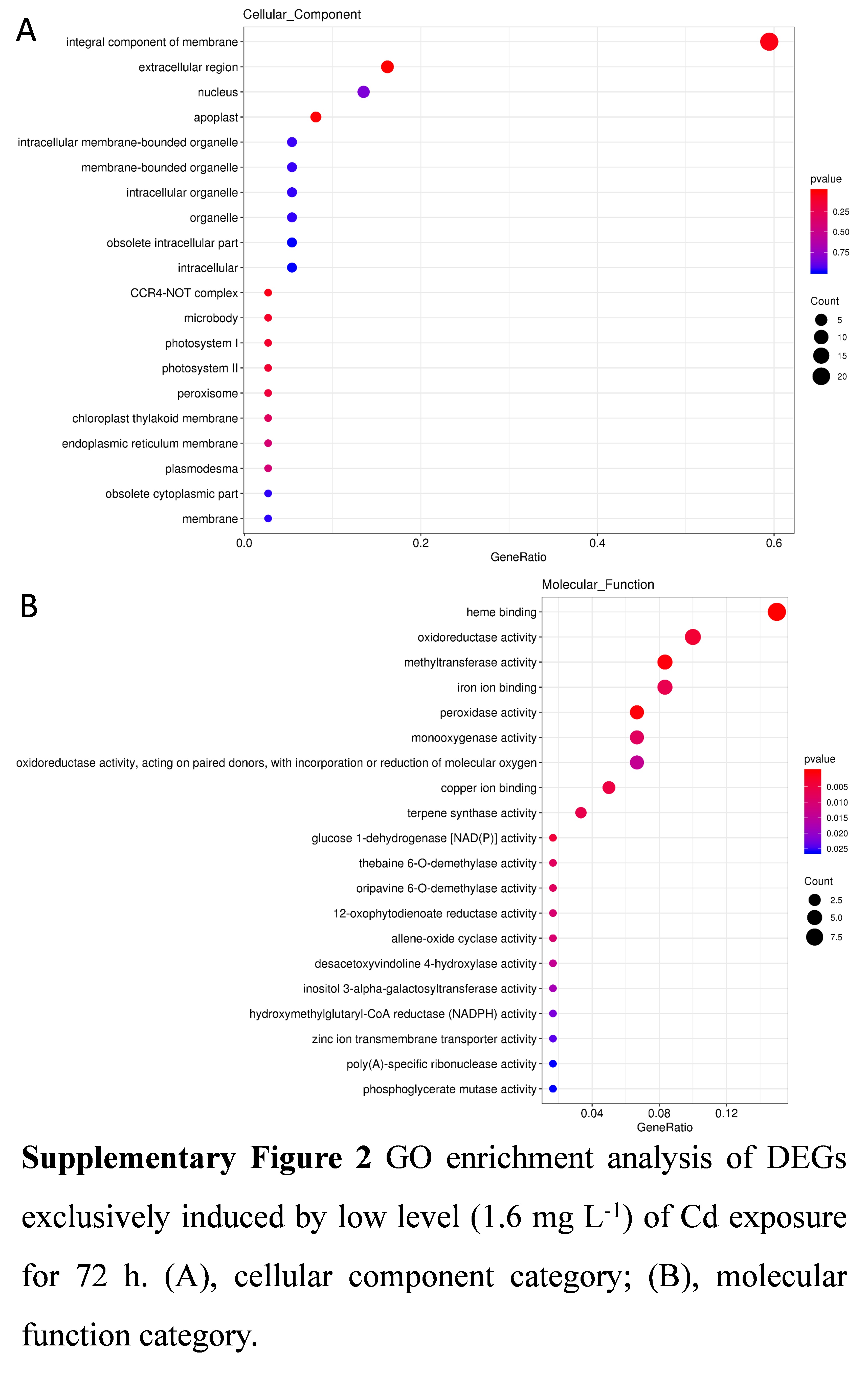

Supplement: Supplementary file 4 [file Image_3.jpeg]
