# Supplementary material for: Low-level cadmium exposure induced hormesis in peppermint young plant by constantly activating antioxidant activity based on physiological and transcriptomic analyses
Source: Front Plant Sci. 2023 Jan 23;14:1088285. doi: 10.3389/fpls.2023.1088285 (PMC9899930; doi:10.3389/fpls.2023.1088285)
Supplement: Supplementary file 5 [file Table_1.doc]

Supplementary Table 1. Primer sequences used in the present study.

| No. | Genes | Forward primer sequence (5'-3') | Reverse primer sequence (5'-3') | Product size (bp) |
| --- | --- | --- | --- | --- |
| 1 | *bHLH47* | ACCCCTATTGTAGGGCCTGT | CAGAGGAAACCTTAGGCGCA | 82 |
| 2 | *bHLH100* | ATTTTCTTGCGGCGATCACG | TATCCCACTCAAGAACGGCG | 141 |
| 3 | *AP2 like* | GCGCCACCGATATCAACAAC | GGCGGAGTTGGTGAGGTAAA | 134 |
| 4 | *WRKY65* | CAAAAGTCCCACACCGGAGA | AGTTGTCCGAAGAGAGCAGC | 70 |
| 5 | *MYB62* | CCTCCCGGAACCAATCAACA | GCTACGGCATGCAAGGATTG | 81 |
| 6 | *JA2L* | AACGATGGGGAAGAGCAAGG | AATGATGGTGGCTCGCTGAA | 70 |
| 7 | *NAC1* | GCCGGAAAAGCTCCAAAAGG | GCGATCGACATTTGCGAGAC | 71 |
| 8 | *WRKY40* | GCCATCTACGAAGGACAGCA | TGCATGTTTTTGGCTCGCTC | 103 |
| 9 | *WRKY51* | AGAGGGCTTCTCTGTCGAGT | CCTAACCCTAGCCGAAACCC | 79 |
| 10 | *WRKY75* | GAAAAGCACCGCATTCCTCC | AAAGGTCATGCCACGAGTGA | 85 |
| 11 | *bZIP61* | TGACGCCATTTTCGATCCCA | ATAATGCCGACACCTCCGAC | 143 |
| 12 | *ZAT10* | GGCTGACCGTGTCCCAATTA | GAGGAGGAAGTCGAAAGCCC | 132 |
| 13 | *ACTIN* | AGCAAAAACAAGCTCTGCCG | TGGAATAGGACCTCAGGGCA | 102 |
