# Supplementary material for: Low-level cadmium exposure induced hormesis in peppermint young plant by constantly activating antioxidant activity based on physiological and transcriptomic analyses
Source: Front Plant Sci. 2023 Jan 23;14:1088285. doi: 10.3389/fpls.2023.1088285 (PMC9899930; doi:10.3389/fpls.2023.1088285)
Supplement: Supplementary file 6 [file Table_2.docx]

Supplementary Table 2. Sequencing reads statistics after filtering.

| Treatment time (h) | Samples | Read Number^@^ | Base Number^#^ | GC Content (%) | ≥Q30 (%)^*^ |
| --- | --- | --- | --- | --- | --- |
| 0 | Control-1 | 19,071,589 | 5,703,663,752 | 49.87 | 93.08 |
|  | Control-2 | 20,220,987 | 6,048,968,126 | 49.88 | 93.95 |
|  | Control-3 | 19,719,773 | 5,902,185,632 | 49.85 | 93.52 |
| 24 | Control-1 | 20,871,028 | 6,246,020,146 | 49.92 | 93.62 |
|  | Control-2 | 20,976,474 | 6,280,884,972 | 50.17 | 92.73 |
|  | Control-3 | 20,157,902 | 6,031,731,366 | 49.71 | 93.89 |
|  | 1.6 mg/L Cd-1 | 23,245,270 | 6,956,883,994 | 49.42 | 93.08 |
|  | 1.6 mg/L Cd -2 | 22,088,194 | 6,604,808,424 | 49.84 | 93.47 |
|  | 1.6 mg/L Cd -3 | 19,128,085 | 5,717,722,940 | 49.62 | 93.48 |
|  | 6.5 mg/L Cd-1 | 20,608,501 | 6,163,459,370 | 49.55 | 93.39 |
|  | 6.5 mg/L Cd -2 | 21,500,272 | 6,437,527,982 | 49.40 | 93.10 |
|  | 6.5 mg/L Cd -3 | 21,221,309 | 6,350,214,762 | 49.58 | 93.36 |
| 72 | Control-1 | 24,588,349 | 7,350,929,452 | 49.83 | 93.50 |
|  | Control-2 | 22,863,099 | 6,839,230,536 | 49.50 | 93.46 |
|  | Control-3 | 23,356,176 | 6,985,343,154 | 49.39 | 93.34 |
|  | 1.6 mg/L Cd-1 | 19,764,100 | 5,908,809,088 | 49.32 | 93.22 |
|  | 1.6 mg/L Cd -2 | 21,609,755 | 6,466,348,534 | 49.08 | 93.32 |
|  | 1.6 mg/L Cd -3 | 19,317,268 | 5,775,863,082 | 49.00 | 93.35 |
|  | 6.5 mg/L Cd-1 | 22,188,000 | 6,636,730,672 | 48.98 | 93.62 |
|  | 6.5 mg/L Cd -2 | 19,932,869 | 5,966,421,584 | 48.89 | 93.25 |
|  | 6.5 mg/L Cd -3 | 20,429,615 | 6,112,722,618 | 49.03 | 93.17 |

^@^the number of paired-end reads in the clean data.

^#^ total base number of clean data.

^*^ base which quality value is greater than or equal to 30 percentage of total clean data.
