# Supplementary material for: Low-level cadmium exposure induced hormesis in peppermint young plant by constantly activating antioxidant activity based on physiological and transcriptomic analyses
Source: Front Plant Sci. 2023 Jan 23;14:1088285. doi: 10.3389/fpls.2023.1088285 (PMC9899930; doi:10.3389/fpls.2023.1088285)
Supplement: Supplementary file 7 [file Table_3.doc]

Supplementary Table 3. Statistics of assembly.

| Length Range (bp) | Transcript | Unigene |
| --- | --- | --- |
| 200-300 | 56,236(15.74%) | 38,022(39.07%) |
| 300-500 | 52,069(14.57%) | 23,281(23.92%) |
| 500-1000 | 77,541(21.70%) | 15,883(16.32%) |
| 1000-2000 | 105,553(29.54%) | 11,576(11.89%) |
| 2000+ | 65,873(18.44%) | 8,567(8.80%) |
| Total Number | 357,272 | 97,329 |
| Total Length | 432,168,986 | 71,455,167 |
| N50 Length | 1,817 | 1,404 |
| Mean Length | 1209.64 | 734.16 |
