# Supplementary material for: Low-level cadmium exposure induced hormesis in peppermint young plant by constantly activating antioxidant activity based on physiological and transcriptomic analyses
Source: Front Plant Sci. 2023 Jan 23;14:1088285. doi: 10.3389/fpls.2023.1088285 (PMC9899930; doi:10.3389/fpls.2023.1088285)
Supplement: Supplementary file 8 [file Table_4.doc]

Supplementary Table 4. Annotated results of unigenes by blasting several public databases.

| Databases | Annotated Number | 300<=length<1000 | length>=1000 |
| --- | --- | --- | --- |
| COG | 10389 | 3050 | 5618 |
| GO | 36205 | 12126 | 14701 |
| KEGG | 28082 | 9029 | 12602 |
| KOG | 23856 | 7705 | 10332 |
| Pfam | 28991 | 9331 | 14293 |
| Swissprot | 26799 | 8508 | 12637 |
| TrEMBL | 42183 | 14227 | 17244 |
| eggNOG | 35070 | 11652 | 15242 |
| nr | 43671 | 14774 | 17267 |
| All annotated | 45360 (46.60%) | 15495 | 17381 |
