# Supplementary material for: Low-level cadmium exposure induced hormesis in peppermint young plant by constantly activating antioxidant activity based on physiological and transcriptomic analyses
Source: Front Plant Sci. 2023 Jan 23;14:1088285. doi: 10.3389/fpls.2023.1088285 (PMC9899930; doi:10.3389/fpls.2023.1088285)
Supplement: Supplementary file 9 [file Table_5.doc]

Supplementary Table 5. Alignment results of each sample.

| Treatment time (h) | Samples | Clean Reads | Mapped Reads | Mapped Ratio (%) |
| --- | --- | --- | --- | --- |
| 0 | Control-1 | 19,071,589 | 12,964,816 | 67.98 |
|  | Control-2 | 20,220,987 | 13,955,199 | 69.01 |
|  | Control-3 | 19,719,773 | 13,584,854 | 68.89 |
| 24 | Control-1 | 20,871,028 | 14,386,833 | 68.93 |
|  | Control-2 | 20,976,474 | 14,031,390 | 66.89 |
|  | Control-3 | 20,157,902 | 13,822,695 | 68.57 |
|  | 1.6 mg/L Cd-1 | 23,245,270 | 15,841,920 | 68.15 |
|  | 1.6 mg/L Cd-2 | 22,088,194 | 15,226,920 | 68.94 |
|  | 1.6 mg/L Cd-3 | 19,128,085 | 13,165,214 | 68.83 |
|  | 6.5 mg/L Cd-1 | 20,608,501 | 14,229,538 | 69.05 |
|  | 6.5 mg/L Cd-2 | 21,500,272 | 14,669,945 | 68.23 |
|  | 6.5 mg/L Cd-3 | 21,221,309 | 14,730,956 | 69.42 |
| 72 | Control-1 | 24,588,349 | 17,047,056 | 69.33 |
|  | Control-2 | 22,863,099 | 15,588,793 | 68.18 |
|  | Control-3 | 23,356,176 | 16,111,227 | 68.98 |
|  | 1.6 mg/L Cd-1 | 19,764,100 | 13,523,627 | 68.43 |
|  | 1.6 mg/L Cd-2 | 21,609,755 | 14,813,426 | 68.55 |
|  | 1.6 mg/L Cd-3 | 19,317,268 | 13,247,275 | 68.58 |
|  | 6.5 mg/L Cd-1 | 22,188,000 | 14,980,767 | 67.52 |
|  | 6.5 mg/L Cd-2 | 19,932,869 | 13,587,033 | 68.16 |
|  | 6.5 mg/L Cd-3 | 20,429,615 | 14,040,236 | 68.72 |
